# The impact of glucagon to support postabsorptive glucose flux and glycemia in healthy rats and its attenuation in male Zucker diabetic fatty rats

**DOI:** 10.1152/ajpendo.00192.2023

**Published:** 2024-01-24

**Authors:** Shanea K. Estes, Chiyo Shiota, Tracy P. O’Brien, Richard L. Printz, Masakazu Shiota

**Affiliations:** ^1^Department of Molecular Physiology and Biophysics, Vanderbilt University School of Medicine, Nashville, Tennessee, United States; ^2^Department of Medicine, Vanderbilt University School of Medicine, Nashville, Tennessee, United States

**Keywords:** glucagon, glucose effectiveness, glucose flux, insulin, type 2 diabetes

## Abstract

Hyperglucagonemia is a hallmark of type 2 diabetes (T2DM), yet the role of elevated plasma glucagon (P-GCG) to promote excessive postabsorptive glucose production and contribute to hyperglycemia in patients with this disease remains debatable. We investigated the acute action of P-GCG to safeguard/support postabsorptive endogenous glucose production (EGP) and euglycemia in healthy Zucker control lean (ZCL) rats. Using male Zucker diabetic fatty (ZDF) rats that exhibit the typical metabolic disorders of human T2DM, such as excessive EGP, hyperglycemia, hyperinsulinemia, and hyperglucagonemia, we examined the ability of hyperglucagonemia to promote greater rates of postabsorptive EGP and hyperglycemia. Euglycemic or hyperglycemic basal insulin (INS-BC) and glucagon (GCG-BC) clamps were performed in the absence or during an acute setting of glucagon deficiency (GCG-DF, ∼10% of basal), either alone or in combination with insulin deficiency (INS-DF, ∼10% of basal). Glucose appearance, disappearance, and cycling rates were measured using [2-^3^H] and [3-^3^H]-glucose. In ZCL rats, GCG-DF reduced the levels of hepatic cyclic AMP, EGP, and plasma glucose (PG) by 50%, 32%, and 50%, respectively. EGP fell in the presence GCG-DF and INS-BC, but under GCG-DF and INS-DF, EGP and PG increased two- and threefold, respectively. GCG-DF revealed the hyperglucagonemia present in ZDF rats lacked the ability to regulate hepatic intracellular cyclic AMP levels and glucose flux, since EGP and PG levels fell by only 10%. We conclude that the liver in T2DM suffers from resistance to all three major regulatory factors, glucagon, insulin, and glucose, thus leading to a loss of metabolic flexibility.

**NEW & NOTEWORTHY** In postabsorptive state, basal plasma insulin (P-INS) and plasma glucose (PG) act dominantly to increase hepatic glucose cycling and reduce endogenous glucose production (EGP) and PG in healthy rats, which is only counteracted by the acute action of basal plasma glucagon (P-GCG) to support EGP and euglycemia. Hyperglucagonemia, a hallmark of type 2 diabetes (T2DM) present in Zucker diabetic fatty (ZDF) rats, is not the primary mediator of hyperglycemia and high EGP as commonly thought; instead, the liver is resistant to glucagon as well as insulin and glucose.

## Graphical abstract

**Figure:**
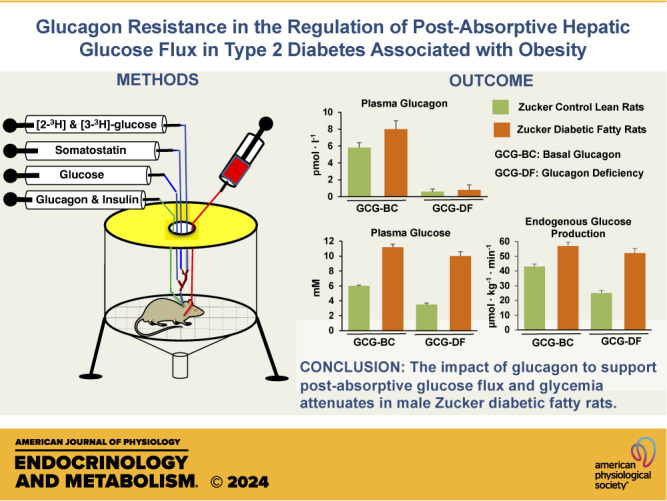

## INTRODUCTION

Postabsorptive plasma glucose (PG) concentration is maintained within a narrow range in healthy subjects by the interplay of the acute glucose-lowering actions of insulin via stimulation of glucose disposal along with inhibition of endogenous glucose production (EGP) and the acute glucose-elevating action of glucagon via stimulation of hepatic glucose production (HGP). As demonstrated by an extensive reduction of PG and EGP levels in response to an acute onset of selective glucagon deficiency (1–4), plasma glucagon (P-GCG) at a basal level is a key supporter of postabsorptive HGP and blood glucose levels in healthy individuals.

Obesity is considered one of the most potent risk factors for type 2 diabetes (T2DM; 5) with up to 85% of people with T2DM also being associated with obesity (6, 7). Postabsorptive hyperglucagonemia is a hallmark of T2DM associated with obesity (T2DM-OB; 8, 9) as well as the pathogenesis of postabsorptive hyperglycemia and raised EGP (10–13). This concept was initially solidified by the glucose-lowering effects of glucagon receptor (GCGR) antagonists in individuals with T2DM (13–16). Recently, however, a solely direct effect of GCGR antagonists to lower plasma glucose by blocking GCG action has arisen with recent reports suggesting an indirect effect of these agents to cause pancreatic α cell hyperplasia leading to increased glucagon-like peptide-1 (GLP-1) levels (14, 16) and/or by promoting proliferation of intestinal L cells and their production and secretion of GLP-1 (17, 18). GLP-1 is well known to have a beneficial effect in the improvement of diabetic hyperglycemia by reducing HGP via a stimulation of glucose-induced insulin secretion from pancreatic β cells (16) and/or via a direct action on the liver (19–22). A GLP-1 receptor blockade markedly attenuated the effect of a GCGR blockade to reduce hyperglycemia and EGP in STZ-induced diabetic mice (23) as well as in db/db mice (24). Therefore, the relative role of a direct blockade of glucagon signaling in the liver and the indirect effects of GCGR blocker treatment leading to an elevation of plasma GLP-1 on the improvement of hyperglycemia in type 2 diabetes remains to be clarified. Thus, the alteration of EGP and glycemia by treatment with GCGR blockers may not provide definitive proof as to whether hyperglucagonemia plays a dominant role in the inappropriately elevated postabsorptive EGP and hyperglycemia in patients with T2DM. An acute setting of selective glucagon deficiency by somatostatin infusion in combination with a basal plasma insulin and glucose clamp, a technique previously utilized for the study of normal healthy subjects (1–4), will allow assessment of the acute role of basal P-GCG but avoids the complicating indirect effects of GCGR blocker treatment and the engagement of compensatory events. So far, however, this type of assessment has not been adapted for individuals and animals with T2DM.

Male Zucker diabetic fatty (ZDF) rats share many metabolic characteristics, such as hyperinsulinemia, hyperglucagonemia, hyperglycemia associated with increased EGP, dyslipidemia, and fatty liver, with individuals with T2DM associated with obesity (25–28). We previously developed a pancreatic hormone and glucose clamp method for rodents (29, 30) wherein endogenous secretion of insulin and glucagon is inhibited by infusing somatostatin, and simultaneously, circulating insulin and glucagon are maintained at their desired levels by the infusion of these hormones into the portal vein. PG levels are also maintained at desired levels by the infusion of glucose into the systemic circulation. This method allows us in rodents to set the plasma concentration of glucagon, insulin, and glucose independently to achieve the desired levels, while maintaining a physiological gradient of these hormones between the portal vein and the systemic circulation. Using the combination of this method and a radioactive tracer method, we investigated the impact of basal P-GCG in the maintenance of postabsorptive glucose flux and euglycemia in healthy rats, as well as the contribution of basal P-GCG to the elevated EGP and hyperglycemia present during the postabsorptive state of ZDF rats.

## MATERIALS AND METHODS

### Animals

Male ZDF rats exhibit obesity and hyperinsulinemia with near normal fasting glycemia when they are younger than 6 wk of age and thereafter develop hyperglycemia with the combination of insulin resistance (25, 31) as well as declining compensatory hyperinsulinemia that is associated with a genetically reduced insulin promoter activity (32), even if they are fed a normal chow diet. These metabolic features bear a resemblance to those in patients with T2DM-OB (25, 33). Unlike their male counterparts, female ZDF rats develop mild hyperglycemia only if they are fed a 60% high-fat diet, and their hyperinsulinemia remains due to hyperplasia of pancreatic β-cells and elevated insulin secretion (31). These female metabolic features resemble those of subjects with obesity. In the present study, therefore, we used only male ZDF rats. Six-week-old male ZDF rats (ZDF/GmiCrl-fa/fa) and their male lean littermates (ZCL: ZD/GmiCrl-+/?) were purchased from Charles River Laboratory, Inc. (Wilmington, MA), fed the 5008 and 5001 Formulab Diet (Purina Mills, St. Louis, MO), respectively, and given water ad libitum in an environmentally controlled room with a 12-h light (from 6:00 AM to 6:00 PM)/12-h dark (from 6:00 PM to 6:00 AM) cycle. Two weeks before each study (at 8 wk of age), rats underwent surgery to place silicon rubber catheters in the left common carotid artery, the right jugular vein, and the ileal vein, as previously described (29, 30, 34, 35). All experiments were conducted in accordance with the Guide for the Care and Use of Laboratory Animals from the National Institute of Health of USA, and all protocols were approved by the Vanderbilt University Institutional Animal Care and Use Committee.

### Plasma Glucagon, Insulin, and/or Glucose Clamp

**Figure 1.**
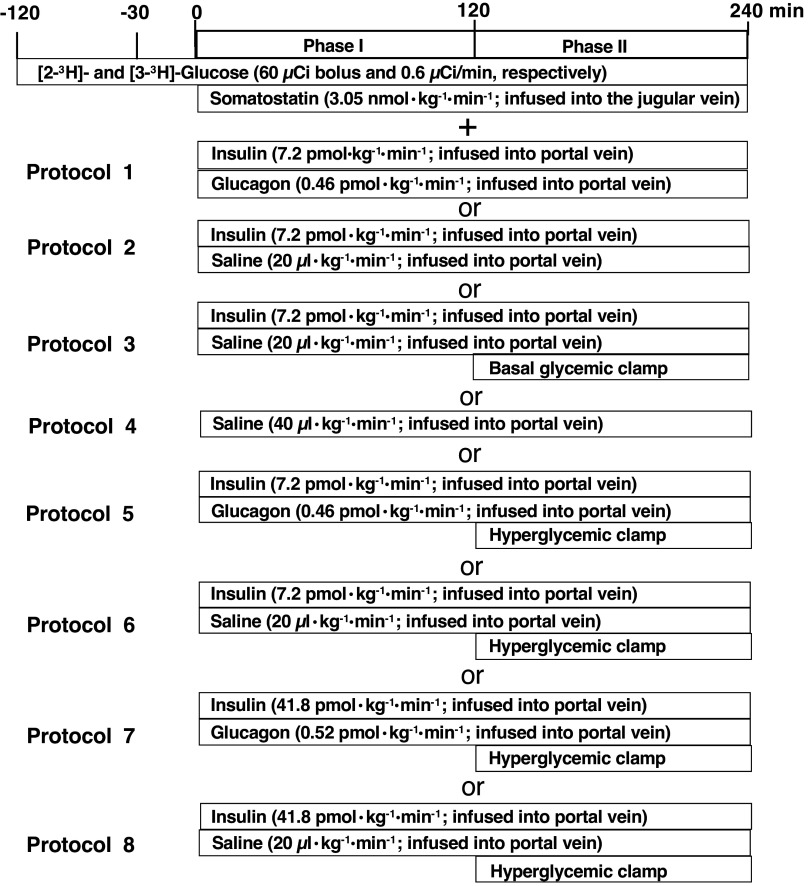
Protocols for basal insulin and/or glucagon clamps (*phase I* and *II*) in Zucker control lean rats (ZCL) (*protocols 1*, *2*, *3*, *4*, *5*, and *6*) and Zucker diabetic fatty (ZDF) (*protocols 7* and *8*) rats. Each clamp study consisted of a 60-min tracer equilibration period (−120 to −30 min), a 30-min basal period (−30 to 0 min), and a 240-min test period (0–240 min). [2-^3^H]- and [3-^3^H]-glucose were given at 60 µCi as a bolus, followed by continuous infusion at 0.6 µCi/min into the systemic circulation. During the test period, somatostatin was infused into the systemic circulation at 3.05 nmol·kg^−1^·min^−1^ through the jugular vein to inhibit endogenous insulin and glucagon secretion in all protocols. To maintain basal plasma levels of insulin in the systemic and portal vein circulation, insulin was infused into the portal vein circulation at 7.2 pmol·kg^−1^·min^−1^ for ZCL rats in *protocols 1*, *2*, *3*, *5*, and *6*, and at 41.8 pmol·kg^−1^·min^−1^ for ZDF rats in *protocols 7* and *8*. When plasma levels of glucagon in the systemic and portal vein circulation were maintained at basal, glucagon was infused into the portal vein circulation at 0.46 pmol·kg^−1^·min^−1^ for ZCL in *protocols 1* and *5*, and at 0.52 pmol·kg^−1^·min^−1^ for ZDF rats in *protocol 7*. To set insulin and/or glucagon deficiency acutely, vehicle (saline), instead of these hormones, was infused into the hepatic portal circulation at 20 µL·kg^−1^·min^−1^ for both ZCL and ZDF rats. During the latter half of the test period (from 120 to 240 min), *phase II*, glucose was infused, if necessary, into the systemic circulation to maintain basal glucose levels for ZCL rats in *protocol 3* and to perform a hyperglycemic clamp at ∼11 mmol/L which corresponds to the basal glucose level of ZDF rats for ZCL rats in *protocols 5* and *6*, and for ZDF rats in *protocols 7* and *8*. We measured glucose flux under a basal condition in ZCL rats in *protocol 1*, changes in glucose flux and glycemia in response to the acute setting of glucagon deficiency in ZCL rats in *protocol 2*, changes in glucose flux in response to acute setting of glucagon deficiency during euglycemic clamp in ZCL rats in *protocol 3*, changes in glucose flux in response to acute setting of a combined deficiency of insulin and glucagon in *protocol 4*, changes in glucose flux in response to acute setting of hyperglycemic clamp in ZCL rats in *protocol 5*, and changes in glucose flux in response to acute setting of glucagon deficiency in the presence of hyperglycemic clamp in ZCL rats in *protocol 6*. In *protocol 7*, insulin and glucagon were infused into the portal vein circulation to maintain the basal plasma insulin and glucagon levels of ZDF rats, and during *phase II*, glucose was infused to maintain basal ZDF plasma glucose levels. In *protocol 8*, only insulin was infused into the portal vein circulation to maintain the basal insulin levels of ZDF rats and allow an acute deficiency in glucagon, and during *phase II*, glucose was infused to maintain basal ZDF plasma glucose levels.

To measure glucose flux in postabsorptive status, animals were fasted from 7:00 AM, and water was given ad libitum until the start of the clamp study. Each clamp study was performed in nonrestricted conscious animals and started from 10:00 AM. As shown in Fig. 1, each clamp study consisted of a 90-min tracer equilibration period (−120 to −30 min), a 30-min basal period (−30 to 0 min), and a 240-min test period (0 to 240 min). To ensure rats were postabsorptive in the basal and test period when glucose flux was measured in the protocols, in a similar group of rats undergoing our basic study protocol, we performed a necropsy between 11:30 AM and 12:00 PM and found that food residual was not present in the stomach, the duodenum, the jejunum, and the ileum, although the cecum was filled with residual and the colon with feces. In addition, in nonrestricted conscious rats catheterized in the carotid artery, and the jugular and the portal veins, we measured the differences in PG concentration and [3-^3^H]-glucose-specific activity between the artery and the portal vein as well as the portal vein and the hepatic vein, and found a negative balance in glucose concentration and no difference in [3-^3^H]-glucose specific activity across the gastrointestinal tract as well as a positive balance in glucose concentration across the liver (data not shown), which indicated no glucose uptake into the circulation from the gastrointestinal tract and glucose production from the liver. Therefore, our feeding regimen includes a sufficient period of fasting to render the animals postabsorptive before starting the measurement of glucose flux in the basal period.

At −120 min in the clamp protocol (Fig. 1), [2-^3^H]- and [3-^3^H]-glucose were given at 60 µCi as a bolus, respectively, followed by continuous infusion at 0.6 µCi/min into the systemic circulation via the jugular vein catheter. During the test period, somatostatin was infused through the jugular vein catheter at 3.05 nmol·kg^−1^·min^−1^ to inhibit endogenous insulin and glucagon secretion in all protocols. When plasma insulin (P-INS) levels were clamped at basal seen in Zucker control lean rats (ZCL) (basal insulin, INS-BC) or ZDF (INS-ZDF-BC) rats, human recombinant Insulin (Novolin R, Novo Nordisk Inc., Plainsboro, NJ) was infused into the portal vein circulation via the ileal vein catheter, at 7.2 pmol·kg^−1^·min^−1^ for ZCL (*protocols 1*, *2*, *3*, *5*, and *6*) and 41.8 pmol·kg^−1^·min^−1^ for ZDF rats (*protocols 7* and *8*). When P-GCG levels were maintained at basal seen in ZCL (GCG-BC) or ZDF (GCG-ZDF-BC) rats, recombinant glucagon (GlucaGen, Novo Nordisk A/S, Bagsvaerd, Denmark) was infused into the portal vein circulation via the ileal vein catheter at 0.46 pmol·kg^−1^·min^−1^ for ZCL (*protocols 1* and *5*) and 0.52 pmol·kg^−1^·min^−1^ for ZDF (*protocol 7*) rats. When glucagon and/or insulin were not infused, vehicle (saline) was substituted for these hormones and infused into the hepatic portal system through the ileal catheter at 20 µL·kg^−1^·min^−1^. During the second half (from 120 to 240 min) of the test period, glucose (50% dextrose solution, Hospira, Inc., Lake Forest, IL) was infused into the systemic circulation through the jugular vein catheter, when clamps were performed, either euglycemic (*protocol 3*) or hyperglycemic (∼11 mM, *protocols 5*, *6*, *7*, and *8*), the basal glycemic level of ZDF rats but hyperglycemia relative to the basal level of ZCL rats. The specific activities of [2-^3^H]- and [3-^3^H]-glucose in plasma glucose were maintained based on our algorism between cold glucose infusion rates and [2-^3^H]- and [3-^3^H]-glucose infusion rates (34).

### Blood and Tissue Collections

Blood samples were collected through the carotid catheter. A sample of 350 µL of blood was collected at 0 min at the end of the basal period and at 60, 120, 180, and 240 min during the test period. A portion (100 µL) of each collected blood sample was immediately deproteinized using perchloric acid for the measurement of lactate and alanine, and the rest of each blood sample was centrifuged to separate plasma. A portion of each plasma sample was deproteinized using Ba(OH)_2_ and ZnSO_4_ for the measurement of glucose and [^3^H]-glucose radioactivity. The rest of each plasma sample was used for the measurement of insulin, glucagon, and free fatty acids (FFAs). A 100 µL blood sample was also collected at −30, 30, 90, 150, and 210 min to measure PG and [^3^H]-glucose radioactivity. At each sampling time, erythrocytes were collected from the blood samples, resuspended in saline, and immediately given back to the animal during the study. In addition, 10 µL of blood was collected once every 10 min during the test period and blood glucose levels were monitored using an Accu-Chek glucometer (Roche, Indianapolis, IN). At the end of the test period, animals were anesthetized by intraarterial infusion of sodium pentobarbital at 50 mg·kg^−1^ through the carotid catheter, and immediately a laparotomy was performed, and the left lobe of the liver was frozen using Wollenberg tongs precooled in liquid nitrogen.

### Measurement of Metabolites and Hormones in Blood, and Protein Levels and Enzyme Activities in Liver

Glycogen synthase and phosphorylase activities in the liver, plasma levels of insulin, glucose, and nonesterified free fatty acids (FFAs), blood levels of lactate and alanine, and [2-^3^H] and [3-^3^H] radioactivity in PG were determined as previously described (34, 35). Glucagon concentration in plasma and cAMP content in liver were measured using a glucagon ELISA kit (Mercodia AB, Sweden) and the cAMP assay kit-direct immunoassay (Abcam, Waltham, MA), respectively.

### Calculations

Glucose fluxes, such as glucose appearance (Ra), glucose disappearance (Rd), total glucose output (glucose 6-phosphatase flux, TGO), EGP, and glucose cycling (GC) rate, were calculated using a one-compartment model with nonsteady-state equations as shown in detail previously (34).

### Statistical Analysis

Results are expressed as means ± SE. Statistical comparison between groups were made by one- or two-way ANOVA, followed by a Student–Newman–Keuls post hoc test, using GraphPad Prism v4 (San Diego, CA). Differences were considered significant at *P* < 0.05.

## RESULTS

### Effects of an Acute Deficiency of Glucagon Alone or Combined with an Insulin Deficiency on Postabsorptive PG Levels and Glucose Flux in Normal Rats

**Figure 2.**
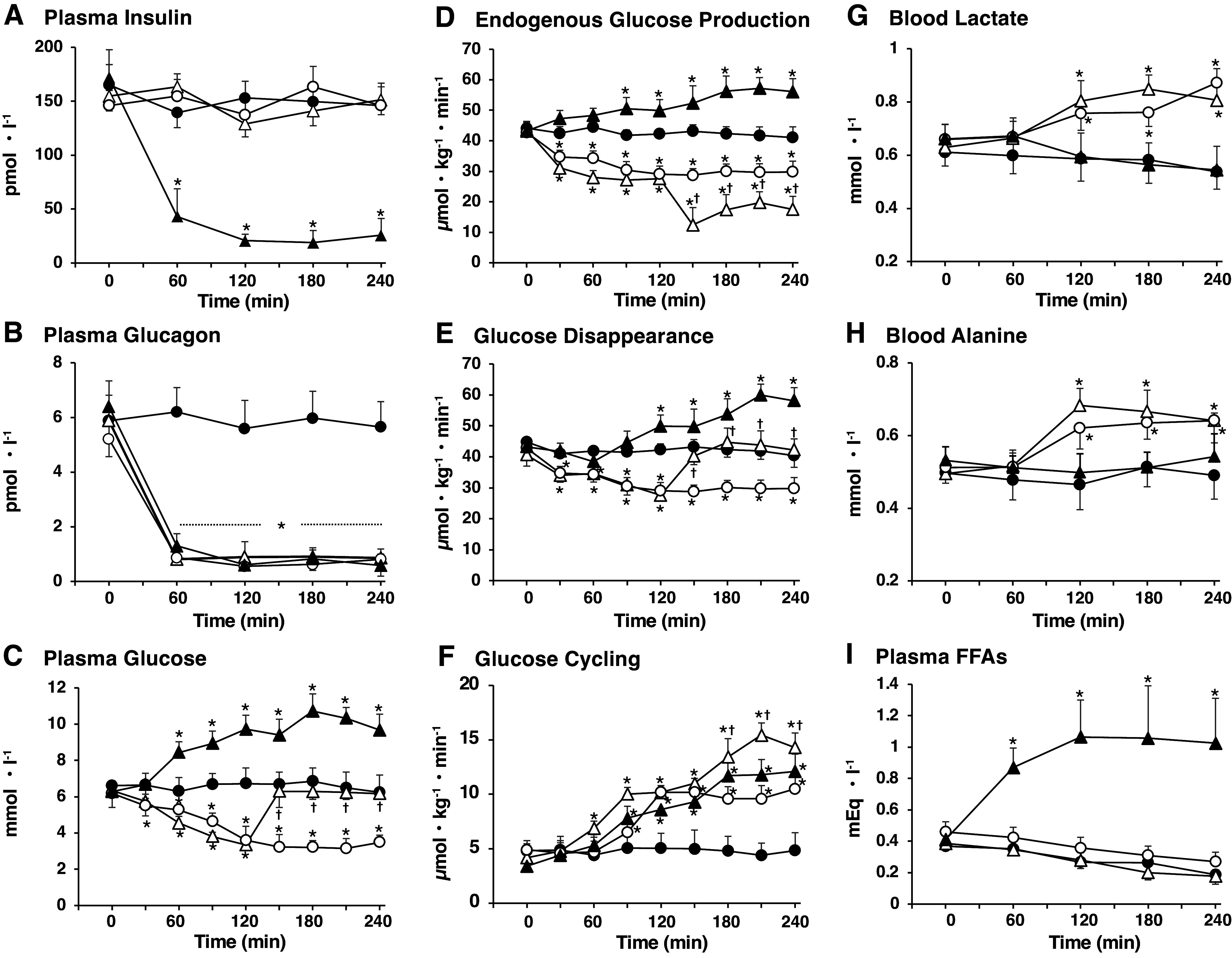
Alterations of glucose flux and blood glucose levels in response to an acute setting of glucagon deficiency in the presence of basal plasma insulin levels and an acute setting of combined insulin and glucagon deficiency in Zucker control lean rats (ZCL) rats. Panels depict plasma insulin (*A*), plasma glucagon (*B*), plasma glucose (*C*), endogenous glucose production (*D*), glucose disappearance (*E*), glucose cycling (*F*), blood lactate (*G*), blood alanine (*H*), and plasma free fatty acids (FFAs) (*I*) under basal and during the test period. The symbols are defined as solid circle, in the presence of basal insulin and glucagon (*protocol 1* in Fig. 1); open circle, under glucagon deficiency (*protocol 2* in Fig. 1); open triangle, under glucagon deficiency with euglycemic clamp during *phase II* (*protocol 3* in Fig. 1); solid triangle, under insulin and glucagon deficiency (*protocol 4* in Fig. 1). Values are means ± SE of six animals in each group. *Significant difference from the corresponding values in the group under the presence of basal glucagon and insulin (*P* < 0.05). †Significant difference from the corresponding values in the group with glucagon deficiency and no clamping of plasma glucose in the presence of basal insulin (*P* < 0.05).

When somatostatin was infused into the systemic circulation simultaneously with insulin and glucagon into the portal vein at the described rates (Fig. 1, *protocol 1*), P-INS, P-GCG, PG, EGP, Rd, GC, and blood lactate and alanine were maintained at basal levels, indicating successful replacement of endogenous insulin and glucagon (Fig. 2 and Table 1). When basal P-INS was replaced alone (Fig. 1, *protocol 2*), P-GCG levels were reduced by 90% within 60 min (Fig. 2*B*). In response to glucagon deficiency (GCG-DF) in the presence of INS-BC (Fig. 2 and Table 2), PG levels were reduced by 50% at 120 min (end of *phase I*) with a 37% reduction of EGP. EGP decreased further by 60% when basal PG levels were restored with glucose infusion during GCG-DF (*phase II*, 120–240 min) in *protocol 3* (Fig. 1) to avoid a counteraction from the lowering of PG concentration against the effect to reduce EGP by GCG-DF itself. These reductions of EGP by GCG-DF were accompanied by a decrease in the EGP fraction contributing to TGO, suggesting a reduction in the supply of glucose 6-phosphate (G-6-P) from an endogenous source. GCG-DF increased GC by twofold in the presence of INS-BC (Fig. 2 and Table 2) and threefold when basal PG was restored (*phase II*, 120–240 min in *protocol 3*, Fig. 1). These increases in GC by GCG-DF were accompanied by a greater GC fraction controlling TGO, which suggests GCG-DF promotes glucose phosphorylation. GCG-DF resulted in elevated levels of blood lactate and alanine, but not plasma FFAs, in either the presence or absence of a euglycemic clamp.

**Table 1.** Alteration of postabsorptive glucose flux in response to acute setting of glucagon alone or with an insulin deficiency in ZCL rats

	0 min (Prior to the Pancreatic Clamp)				120 min (At End of *Phase I* during Test Period)				240 min (At End of *Phase II* during Test Period)			
*Protocol No.*	*1*	*2*	*3*	*4*	*1*	*2*	*3*	*4*	*1*	*2*	*3*	*4*
Clamp Condition	No Clamp	No Clamp	No Clamp	No Clamp	INS-BC GCG-BC	INS-BC GCG-DF	INS-BC GCG-DF	INS-DF GCG-DF	INS-BC GCG-BC	INS-BC GCG-DF	INS-BC GCG-DF PG-BC	INS-DF GCG-DF
Plasma insulin, pmol·L^−1^	182 ± 18	146 ± 14	155 ± 14	172 ± 26	153 ± 15	138 ± 12	129 ± 12	21 ± 7*	146 ± 9	146 ± 21	151 ± 12	26 ± 9*
Plasma glucagon, pmol·L^−1^	5.88 ± 0.93	5.20 ± 0.65	5.88 ± 0.83	6.40 ± 0.95	5.60 ± 1.03	0.55 ± 0.11*	0.89 ± 0.57*	0.60 ± 0.26*	5.65 ± 0.92	0.80 ± 0.20*	0.86 ± 0.32*	0.57 ± 0.40*
Plasma glucose, mmol·L^−1^	6.61 ± 0.27	6.18 ± 0.78	6.34 ± 0.08	6.28 ± 0.55	6.72 ± 0.86	3.59 ± 0.77*	3.34 ± 0.20*	9.72 ± 0.77*	6.24 ± 0.95	3.48 ± 0.40*	6.17 ± 0.20	9.67 ± 0.88*
GIR, µmol·kg^−1^·min^−1^	0	0	0	0	0	0	0	0	0	0	22.7 ± 6.2	0
EGP, µmol·kg^−1^·min^−1^	44.1 ± 1.0	42.8 ± 2.0	43.6 ± 2.8	43.2 ± 2.8	42.3 ± 1.6	29.1 ± 2.6*	27.6 ± 1.6*	49.9 ± 3.6	41.1 ± 3.5	29.8 ± 3.5*	17.6 ± 4.2*	56.1 ± 4.2*
Rd, µmol·kg^−1^·min^−1^	44.8 ± 1.1	42.8 ± 2.0	40.6 ± 3.6	43.2 ± 2.8	42.2 ± 2.0	29.1 ± 2.6*	27.6 ± 1.6*	49.9 ± 3.6	41.4 ± 3.8	29.8 ± 3.5*	42.2 ± 3.6	58.1 ± 4.2*
GC, µmol·kg^−1^·min^−1^	4.8 ± 0.6	4.9 ± 0.9	4.2 ± 0.5	3.4 ± 1.1	5.1 ± 1.4	11.4 ± 1.3*	10.2 ± 0.6*	8.6 ± 0.1*	4.9 ± 1.6	10.5 ± 1.3*	14.3 ± 1.3*	12.1 ± 1.6*
TGO, µmol·kg^−1^·min^−1^	48.9 ± 1.4	47.7 ± 2.3	47.8 ± 2.7	46.6 ± 2.3	47.4 ± 2.6	40.5 ± 3.0*	37.8 ± 2.1*	58.5 ± 3.4*	46.0 ± 3.3	40.3 ± 2.6*	31.9 ± 3.8*	68.2 ± 3.8*
EGP fraction (%) in TGO	90.2 ± 3.3	89.7 ± 4.1	91.2 ± 3.8	92.7 ± 3.4	89.2 ± 3.2	71.9 ± 5.9	73.0 ± 4.2	85.3 ± 4.3	89.3 ± 4.1	73.9 ± 4.9*	55.2 ± 10.3*	82.3 ± 5.7
GC fraction (%) in TGO	9.8 ± 3.3	10.3 ± 4.1	8.8 ± 3.8	7.3 ± 3.4	10.8 ± 3.2	28.1 ± 5.9*	27.0 ± 4.2*	14.7 ± 4.3	10.7 ± 4.1	26.1 ± 4.9*	44.8 ± 10.3*	17.7 ± 5.7
Blood lactate, mmol·L^−1^	0.61 ± 0.06	0.66 ± 0.06	0.63 ± 0.07	0.66 ± 0.06	0.59 ± 0.08	0.77 ± 0.06*	0.80 ± 0.08*	0.59 ± 0.09	0.54 ± 0.07	0.87 ± 0.05*	0.81 ± 0.06*	0.54 ± 0.09
Blood alanine, mmol·L^−1^	0.50 ± 0.03	0.51 ± 0.02	0.50 ± 0.03	0.53 ± 0.04	0.47 ± 0.07	0.62 ± 0.06*	0.68 ± 0.05*	0.50 ± 0.05	0.49 ± 0.07	0.64 ± 0.06	0.64 ± 0.02*	0.54 ± 0.06
Plasma FFAs, meq·L^−1^	0.37 ± 0.02	0.46 ± 0.07	0.39 ± 0.02	0.41 ± 0.05	0.27 ± 0.04	0.36 ± 0.07	0.28 ± 0.05	1.06 ± 0.24*	0.19 ± 0.03	0.27 ± 0.06	0.18 ± 0.05	1.03 ± 0.29*

Values are means ± SE of six experiments. EGP, endogenous glucose production; EGP fraction in TGO, the percentage of glucose produced from G6P derived from endogenous source(s), glycogenolysis and/or gluconeogenesis, in TGO; GC, glucose cycling; GCG-BC, glucagon clamp at basal; GCG-DF, acute setting of glucagon deficiency; GC fraction in TGO, the percentage of glucose produced from G6P derived from plasma glucose in TGO; GIR, glucose infusion rate; INS-BC, insulin clamp at basal; INS-DF, acute setting of insulin deficiency; PG-BC, glucose clamp at basal; Rd, glucose disappearance; TGO, total glucose output; ZCL, Zucker control lean rats. *Significant difference (*P* < 0.05) from values of the *protocol 1* at the corresponding time point.

**Table 2.** Alteration of postabsorptive glucose flux in response to acute setting of glucagon deficiency and hyperglycemia in ZDF rats

	0 min (Prior to the Pancreatic Clamp)				120 min (during the Pancreatic Clamp)				240 min (during the Pancreatic Clamp)			
Animals	ZCL		ZDF		ZCL		ZDF		ZCL		ZDF	
*Protocol No.*	*5*	*6*	*7*	*8*	*5*	*6*	*7*	*8*	*5*	*6*	*7*	*8*
Clamp Condition	No Clamp	No Clamp	No Clamp	No Clamp	INS-BC GCG-BC	INS-BC GCG-DF	INS-ZDF-BC GCG-ZDF-BC	INS-ZDF-BC GCG-DF	INS-BC GCG-BC PG-ZDF-HC	INS-BC GCG-DF PG-ZDF-HC	INS-ZDF-BC GCG-ZDF-BC PG-ZDF-HC	INS-ZDF-BC GCG-DF PG-ZDF-HC
Plasma insulin, pmol·L^−1^	182 ± 19	155 ± 14	1,118 ± 120*	1,049 ± 103†	153 ± 15	129 ± 12	1,100 ± 120*	1,049 ± 86†	163 ± 9	143 ± 12	1,066 ± 52*	1,032 ± 155†
Plasma glucagon, pmol·L^−1^	5.40 ± 0.86	5.77 ± 0.63	7.98 ± 0.95*	7.26 ± 0.98†	6.11 ± 0.83	0.63 ± 0.34*	8.87 ± 0.66*	0.83 ± 0.60†	5.97 ± 1.00	0.77 ± 0.37*	8.18 ± 0.34*	0.46 ± 0.43‡
Plasma glucose, mmol·L^–1^	6.10 ± 0.20	6.18 ± 0.16	11.7 ± 0.8*	12.2 ± 0.5†	5.96 ± 0.14	3.52 ± 0.16*	11.2 ± 0.4*	10.0 ± 0.6†	11.3 ± 0.4	11.5 ± 0.2	11.3 ± 0.4	11.4 ± 0.8
GIR, µmol·kg^−1^·min^−1^	0	0	0	0	0	0	0	0	52.8 ± 5.1	72.6 ± 11.8	7.6 ± 5.3*	23.0 ± 6.2†
EGP, µmol·kg^−1^·min^−1^	44.1 ± 1.2	44.5 ± 1.5	59.2 ± 2.9*	58.6 ± 2.2†	43.0 ± 1.7	25.0 ± 1.8*	57.9 ± 2.7*	52.1 ± 3.1†	25.0 ± 5.0	4.4 ± 2.5*	50.6 ± 5.2*	40.2 ± 2.6†‡
Rd, µmol·kg^−1^·min^−1^	44.2 ± 1.1	44.5 ± 1.5	55.5 ± 4.9*	54.8 ± 4.8†	43.0 ± 1.7	27.5 ± 1.9*	56.5 ± 4.6*	54.5 ± 6.2†	80.3 ± 3.0	75.2 ± 9.0	58.0 ± 4.4*	65.8 ± 7.4
GC, µmol·kg^−1^·min^−1^	5.9 ± 1.3	5.1 ± 2.0	28.5 ± 5.6*	25.9 ± 4.2†	6.2 ± 0.8	11.9 ± 3.2*	30.1 ± 6.5*	33.9 ± 3.9†	17.7 ± 2.3	55.5 ± 6.8*	30.8 ± 6.0*	44.1 ± 4.6†‡
TGO, µmol·kg^−1^·min^−1^	50.0 ± 1.3	49.6 ± 1.9	87.7 ± 5.1*	84.5 ± 4.8†	49.2 ± 1.9	36.9 ± 3.1*	88.0 ± 4.2*	86.0 ± 3.3†	42.7 ± 4.2	59.9 ± 5.3*	81.4 ± 4.8*	84.3 ± 5.2†
EGP fraction (%) in TGO	88.2 ± 3.2	89.7 ± 3.3	67.5 ± 4.3*	68.8 ± 3.1†	87.4 ± 3.2	67.8 ± 7.6*	65.8 ± 5.1*	60.6 ± 4.2	58.8 ± 6.7	7.3 ± 4.3*	62.2 ± 3.9	47.7 ± 4.7†‡
GC fraction (%) in TGO	11.8 ± 3.1	10.3 ± 3.3	32.5 ± 4.3*	31.2 ± 3.1†	12.6 ± 3.2	32.2 ± 7.6*	34.2 ± 5.1*	39.4 ± 4.2	41.2 ± 6.7	92.7 ± 4.3*	37.8 ± 3.9	52.3 ± 4.7†‡
Blood lactate, mmol·L^−1^	0.46 ± 0.05	0.43 ± 0.04	1.39 ± 0.16*	1.39 ± 0.17†	0.46 ± 0.04	0.56 ± 0.04	1.51 ± 0.21*	1.65 ± 0.30†	0.64 ± 0.06	0.52 ± 0.03	1.50 ± 0.24*	1.78 ± 0.18†
Blood alanine, mmol·L^−1^	0.48 ± 0.01	0.53 ± 0.03	0.57 ± 0.05	0.56 ± 0.05	0.49 ± 0.08	0.63 ± 0.06*	0.48 ± 0.03	0.51 ± 0.04	0.42 ± 0.01	0.66 ± 0.04*	0.44 ± 0.03	0.41 ± 0.04†
Plasma FFAs, meq·L^−1^	0.46 ± 0.08	0.39 ± 0.02	1.70 ± 0.24*	1.92 ± 0.28†	0.45 ± 0.09	0.38 ± 0.03	1.69 ± 0.28*	2.21 ± 0.19†	0.18 ± 0.04	0.21 ± 0.03	1.79 ± 0.39*	1.96 ± 0.16†

Values are means ± SE of six experiments for Zucker control lean (ZCL) rats and eight experiments for Zucker diabetic fatty (ZDF) rats. EGP, endogenous glucose production rate; EGP fraction in TGO, the percentage of glucose produced from glucose-6-phosphate derived from endogenous source(s), glycogenolysis and/or gluconeogenesis, in TGO; FFA, free fatty acid; GC, glucose cycling rate; GC fraction in TGO, the percentage of glucose produced from glucose-6-phosphate derived from plasma glucose in TGO; GCG-BC, glucagon clamp at basal in ZCL; GCG-DF, acute setting of glucagon deficiency; GCG-ZDF-BC, glucagon clamp at basal in ZDF; GIR, glucose infusion rate; INS-BC, insulin clamp at basal in ZCL; INS-DF, acute setting of insulin deficiency; INS-ZBC, insulin clamp at basal in ZDF rats; PG-ZDF-HC, hyperglycemic clamp at basal levels seen in ZDF; Rd, glucose disappearance rate; TGO, total glucose output rate. Significant difference (*P* < 0.05) from values of the *protocol 5, the †*protocol 6*, and the ‡*protocol 7* at the corresponding time point.

**Figure 3.**
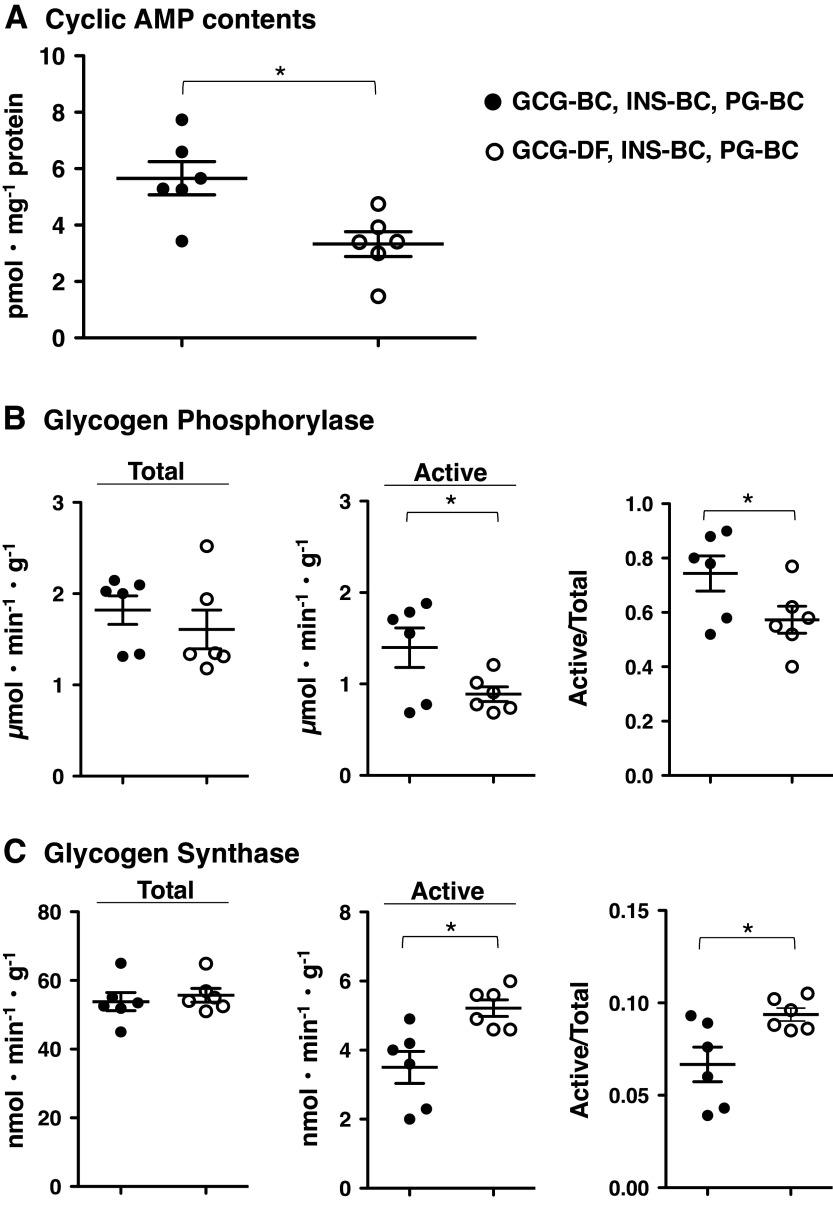
Effects of acute setting of glucagon deficiency on hepatic cyclic AMP content, glycogen phosphorylase activity, and glycogen synthase activity in Zucker control lean rats (ZCL) rats. The livers were collected at the end of the test period in each protocol. Panels depict: hepatic cyclic AMP content (*A*), total activity of glycogen phosphorylase, activity of the active form of glycogen phosphorylase and the ratio of activity of the active form to total activity (*B*), and activity of total glycogen synthase, activity of the active form of glycogen synthase and the ratio of activity of the active form to total phosphorylase activity (*C*). The symbols are defined as solid circle, in the presence of basal insulin and glucagon (+G; *protocol 1* in Fig. 1); and open circle, under glucagon deficiency (−G; *protocol 2* in Fig. 1). Values are means ± SE of six animals in each group. *Significant difference from the corresponding values in the group under the presence of basal glucagon and insulin (*P* < 0.05). GCG-BC, glucagon clamp at the basal plasma glucagon of ZCL rats; GCG-DF, glucagon deficiency; INS-BC, insulin clamp at the basal plasma insulin of ZCL rats; PG, plasma glucose.

A part (50–60%) of postabsorptive HGP is due to glycogenolysis (36). As shown in Fig. 3, in response to GCG-DF under INS-BC and PG-BC (Fig. 1, *protocol 3*), compared with the presence of INS-BC and GGN-BC (Fig. 1, *protocol 1*), hepatic cAMP levels, the activity of the active form (GPa) of glycogen phosphorylase, and the ratio of GPa to total GP activity (GPa+b) decreased by 40%. In contrast, the activity of the active form (GSi) of glycogen synthase (GS) and the ratio of GS_I_ to total GS activity (GSi+d) were elevated by 45%. These results suggest that supporting postabsorptive HGP by basal glucagon is, at least in part, associated with sustaining basal levels of intracellular cAMP and glycogenolysis.

To measure the impact of basal INS on postabsorptive glucose flux, acute insulin deficiency (INS-DF) was performed under GCG-DF (Fig. 1, *protocol 4*). As shown in Fig. 2 and Table 1, with the initiation of somatostatin infusion, P-INS and P-GCG declined promptly to ∼10% of their basal levels. In response to the combination of INS-DF and GCG-DF, EGP and PG levels were elevated two- and threefold, respectively. The elevation of EGP was associated with a 1.6-fold increase in the TGO without a change in the EGP fraction contributing to TGO, suggesting increased provision of G-6-P from an endogenous source. On the other hand, GC did not increase significantly despite a threefold increase in PG concentration. Thus, INS-DF appears to lead to a suppression of glucose-induced GC that could then contribute to hyperglycemia by increasing EGP. Since INS-DF also increased plasma FFAs levels, an increase in EGP induced by INS-DF might be partially mediated by the elevation of plasma FFAs (37).

### Effect of GCG-DF on Postabsorptive Plasma Glucose Levels and Glucose Flux in ZDF Rats

In ZDF rats, when glucagon and insulin were infused intraportally with the systemic infusion of somatostatin (Fig. 1, *protocols 7* and *8*), metabolic features of T2DM-OB seen during the basal period, such as hyperinsulinemia, hyperglucagonemia, hyperglycemia, hyperlactatemia, hyperlipidemia, and greater rates of TGO, EGP, and GC compared with that of ZCL rats, were maintained, indicating successful replacement of their endogenous insulin and glucagon by the intraportal infusion of these hormones at the indicated infusion rates. In ZCL rats, in response to GCG-DF alone at 120 min during the test period (Fig. 1, *protocol 6*), compared with that at the same time point under GCG-BC, PG concentrations and EGP decreased by 43% (−Δ 2.66 mM) and 44% (−Δ 19.5 µmol·kg^−1^·min^−1^), respectively, with a decrease in TGO by 25% (−Δ 12.3 mol·kg^−1^·min^−1^) and in the EGP fraction contributing to TGO from 87% under GCG-BC to 68% (−Δ 21.9 µmol·kg^−1^·min^−1^) with GCG-DF. In contrast, GC increased by twofold (+Δ 6.8 µmol·kg^−1^·min^−1^) and the GC fraction contributing to TGO by 2.5-fold (32%). Blood lactate and alanine levels increased significantly by 22% and 29%, respectively. In ZDF rats, on the other hand, during *phase I* with GCG-ZDF-BC and INS-ZDF-BC (Fig. 1, *protocol 7*), despite sustained hyperglycemia at basal levels, PG and EGP only tended to show a small decrease by 4.3% (−Δ 0.5 mM) and by 2.2% (−Δ 1.3 µmol·kg^−1^·min^−1^), respectively, and GC likewise only tended to increase by 5.6% (+Δ1.6 µmol·kg^−1^·min^−1^), although these alterations are not statistically significant. In response to GCG-DF (Fig. 1, *protocol 8*), compared with that in the presence of GCG-ZDF-BC at 120 min, PG and EGP decreased by 11% (−Δ 1.2 mM) and 10% (−Δ 5.8 µmol·kg^−1^·min^−1^), respectively, whereas GC was increased by 13% (+Δ 2.8 µmol·kg^−1^·min^−1^). Both the percentage and absolute rate of decrease of EGP as well as increase of GC with GCG-DF relative to GCG-BC in ZDF rats were markedly smaller compared with that in ZCL rats.

To compare the effect of GCG-DF on glucose flux between ZCL and ZDF rats in the presence of equivalent plasma glucose concentrations, PG levels were matched by using a hyperglycemic clamp at the basal PG level of ZDF rats (PG-ZDF-HC) during *phase II* of the test period in ZCL (Fig. 1, *protocols 5* and *6*) and ZDF (Fig. 1, *protocols 7* and *8*) rats. In ZDF rats, during *phase II* when basal hyperglycemia was restored with GCG-ZDF-BC and INS-ZDF-BC, EGP and TGO tended to reduce progressively by 13% (−Δ 7.3 µmol·kg^−1^·min^−1^) and 7% (−Δ 6.6 µmol·kg^−1^·min^−1^), respectively, with a slight decrease in the EGP fraction contributing to TGO from 66% to 62%. GC and the GC fraction contributing to TGO remained at their basal levels. In ZCL rats, when PG concentration was clamped at PG-ZDF-HC, EGP decreased by 42% (−Δ 18 µmol·kg^−1^·min^−1^) along with a decrease in the EGP fraction contributing to TGO from 87% to 58% in the absence of a significant change in TGO under GCG-BC and INS-BC. As a result, the difference in EGP and GC between ZCL and ZDF rats is larger and smaller, respectively, thus showing a decreased EGP and increased GC in ZCL rats when PG levels are matched at the basal PG level of ZDF rats (PG-ZDF-HC) compared with when PG levels are unmatched.

**Figure 4.**
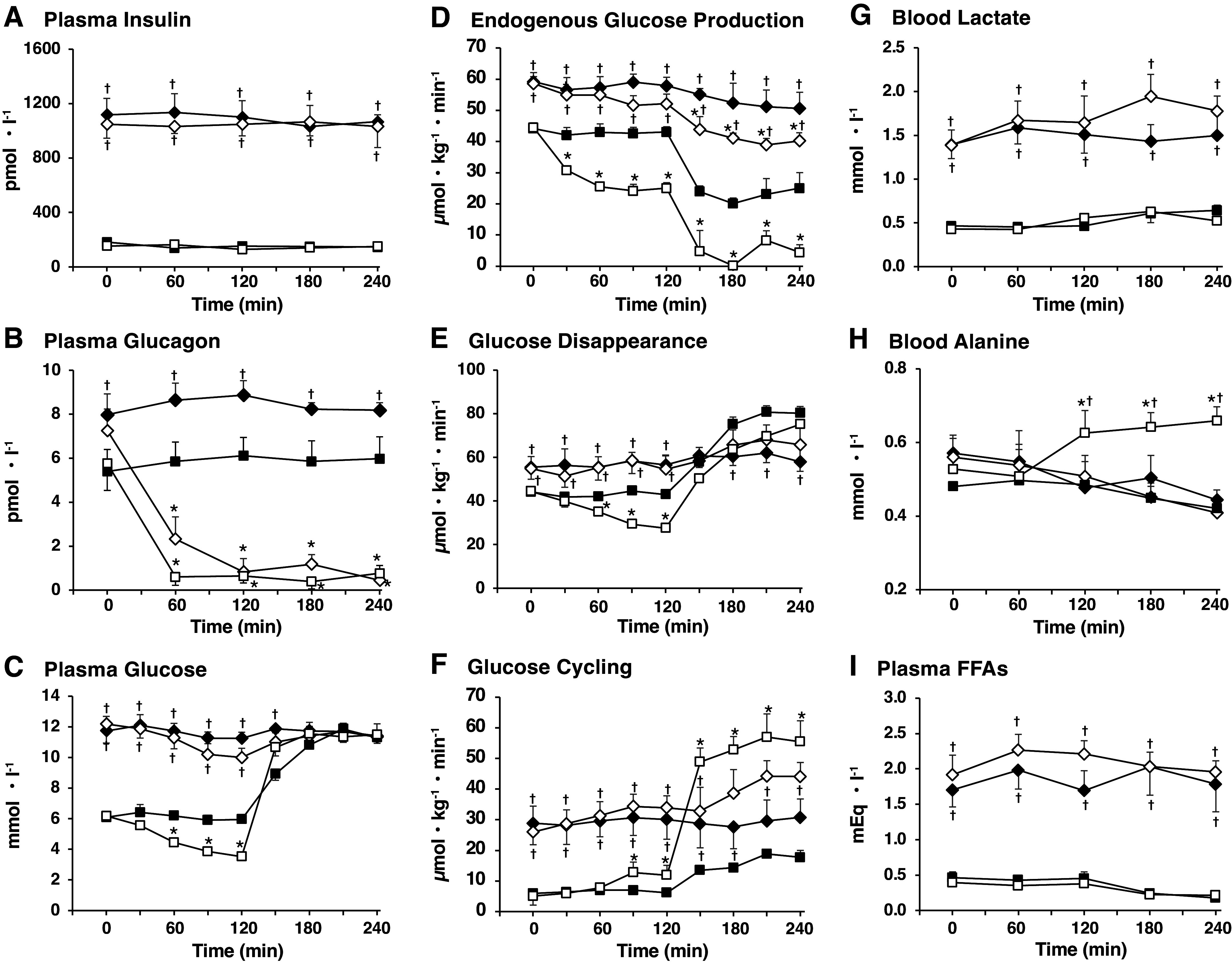
Alterations of glucose flux and blood glucose levels in response to an acute setting of glucagon deficiency and/or hyperglycemic clamp in the presence of basal plasma insulin levels in Zucker control lean rats (ZCL) and Zucker diabetic fatty (ZDF) rats. Panels depict: plasma insulin levels (*A*), plasma glucagon levels (*B*), plasma glucose levels (*C*), endogenous glucose production (*D*), glucose disappearance (*E*), glucose cycling (*F*), blood lactate (*G*), blood alanine (*H*), and plasma free fatty acids (FFAs) (*I*). Metabolic parameters were measure under basal conditions (0 min) and during a basal insulin clamp (Fig. 1, *phase I*, 0–120 min) and basal insulin and basal glucose clamp of ZDF rats (hyperglycemic for ZCL rats; Fig. 1, *phase II*, 120–240 min), in the presence of basal glucagon (Fig. 1, *protocol 5* for ZCL rats and *protocol 7* for ZDF rats) and with glucagon deficiency (Fig. 1, *protocol 6* for ZCL rats and *protocol 8* for ZDF rats). The symbols are defined as solid square, ZCL rats in basal glucagon clamp; open square, ZCL rats in glucagon deficiency; solid diamond, ZDF rats in basal glucagon clamp; and open diamond, ZDF rats in glucagon deficiency. Values are means ± SE of six and eight animals in each group of ZCL and ZDF rats, respectively. *Significant difference from the corresponding values of the basal glucagon group in the identical animal model (*P* < 0.05). †Significant difference from the corresponding values of ZCL rats with the identical glucagon status (*P* < 0.05).

In response to an acute setting of GCG-DF during *phase II* of the test period in ZCL rats, EGP was suppressed completely (−Δ 21 µmol·kg^−1^·min^−1^) with a reduction in the EGP fraction contributing to TGO from 68% to 7% whereas TGO increased nearly twofold, while GC increased threefold (Δ 44 µmol·kg^−1^·min^−1^) along with a threefold increase in the GC fraction contributing to TGO (from 32 to 93%) (Fig. 4, Table 2). Even under equivalent hyperglycemia, therefore, both the percentage and absolute rate of decrease of EGP (20% and −Δ 10 µmol·kg^−1^·min^−1^, respectively) and increase of GC (33% and Δ 11 µmol·kg^−1^·min^−1^, respectively) with GCG-DF compared with GCG-BC in ZDF rats were markedly smaller relative to those in ZCL rats. Thereby, even under equivalent hyperglycemia, the reduction in EGP by GCG-DF was also smaller in ZDF rats than in ZCL rats. These results suggest a limited contribution of basal P-GCG toward abnormally increased postabsorptive EGP and hyperglycemia in ZDF rats.

### Effect of GCG-DF on Hepatic Cyclic AMP Levels and Glycogen Metabolism in ZDF Rats

**Figure 5.**
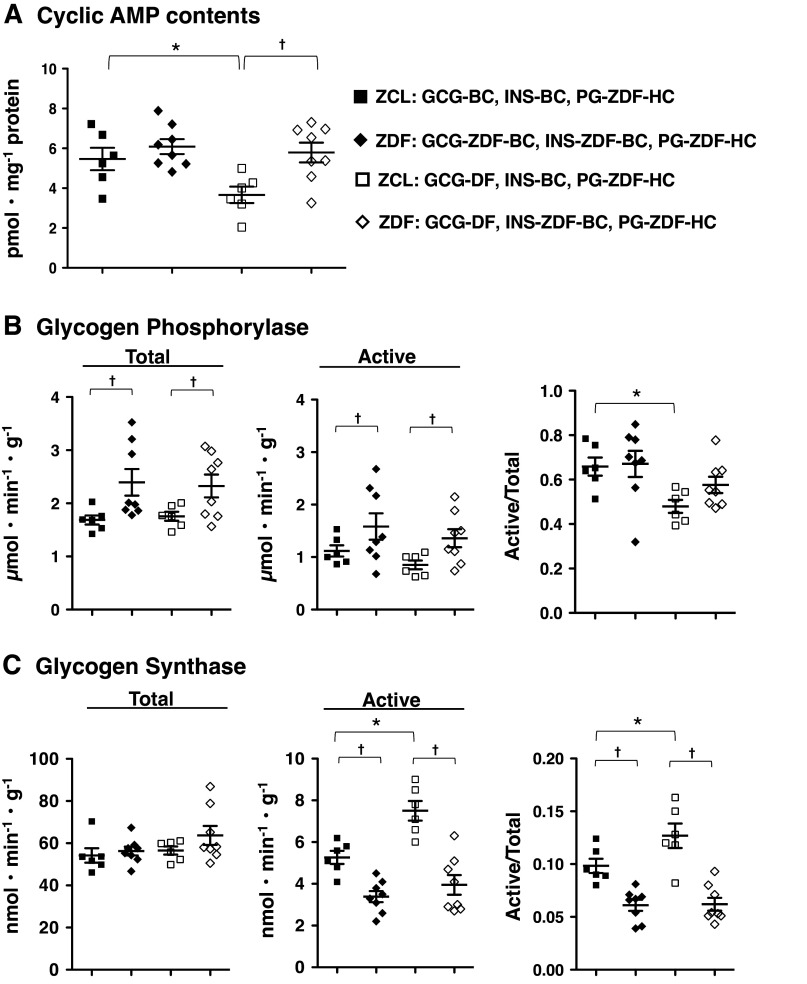
Effects of acute setting of glucagon deficiency on hepatic cyclic AMP content, glycogen phosphorylase activity, and glycogen synthase activity in Zucker control lean rats (ZCL) and Zucker diabetic fatty (ZDF) rats. Animals were fasted for 5 h before each study. The livers were collected at the end of the test period in each protocol. Panels depict: hepatic cyclic AMP content (*A*), total activity of glycogen phosphorylase, the activity of the active form of glycogen phosphorylase and the ratio of activity of active form to total activity (*B*), and total activity of glycogen synthase, the activity of the active form of glycogen synthase and the ratio of activity of the active form to total phosphorylase activity (*C*). The symbols are defined as solid square, ZCL rats in basal glucagon clamp (+G; Fig. 1, *protocol 5*); open square, ZCL rats in glucagon deficiency (–G; Fig. 1, *protocol 6*); solid diamond, ZDF rats in basal glucagon clamp (+G; Fig. 1. *protocol 7*); and open diamond, ZDF rats in glucagon deficiency (–G; Fig. 1, *protocol 8*). Values are means ± SE of six animals in each group of ZCL rats and eight animals in each group of ZDF rats. *Significant difference from the corresponding values of the basal glucagon group in the identical animal model (*P* < 0.05). †Significant difference from the corresponding values of ZCL rats in the identical glucagon status (*P* < 0.05). GCG-BC, glucagon clamp at the basal plasma glucagon of ZCL rats; GCG-DF, glucagon deficiency; GCG-ZDF-BC, glucagon clamp at the basal plasma glucagon of ZDF rats; INS-BC, insulin clamp at the basal plasma insulin of ZCL rats; INS-ZDF-BC, insulin clamp at the basal plasma insulin of ZDF rats; PG-ZDF-HC, hyperglycemic clamp at the basal plasma glucose level of ZDF rats.

Under the combination of PG-ZDF-BC with basal glucagon and insulin (Fig. 1, *protocol 5* in ZCL and *protocol 7* in ZDF rats), hepatic cAMP levels in ZDF rats were not significantly different from those of ZCL rats (Fig. 5), despite higher P-GCG levels in ZDF rats compared with ZCL rats. On the other hand, GPa+b and GPa were higher in ZDF rats compared with those of ZCL rats, although the ratios of GPa to GPa+b activities were similar in both groups. GSi+d did not differ between ZDF and ZCL rats, whereas GSi and the ratio of GSi to GSi+d activities were lower in ZDF rats compared with ZCL rats. These results indicate a profile of glycogen metabolism that favors glycogenolysis in ZDF rats. Comparing these parameters in the presence of basal glucagon to GCG-DF with the combination of hyperglycemia under basal insulin (ZCL rats; Fig. 1, *protocol 6* and ZDF rats; Fig. 1, *protocol 8*), all measured parameters in GCG-DF ZDF rats were not significantly different from those under GCG-ZDF-BC (Fig. 5). Whereas in ZCL rats, cAMP content and the ratios of GPa to GPa+b activities were lower, whereas the activity of GSi and the ratio of GSi to GSi+d activities were higher with GGN-DF. Overall, these results suggest an attenuated regulation of the cAMP signaling pathway by basal P-GCG in ZDF rats; wherein, GCG-DF failed to result in lower hepatic cAMP levels and a switch from a profile favoring glycogenolysis to glycogen synthesis.

### Effects of GCG-DF and/or INS-DF on the Effect of PG on EGP and Rd in ZCL and ZDF Rats

**Figure 6.**
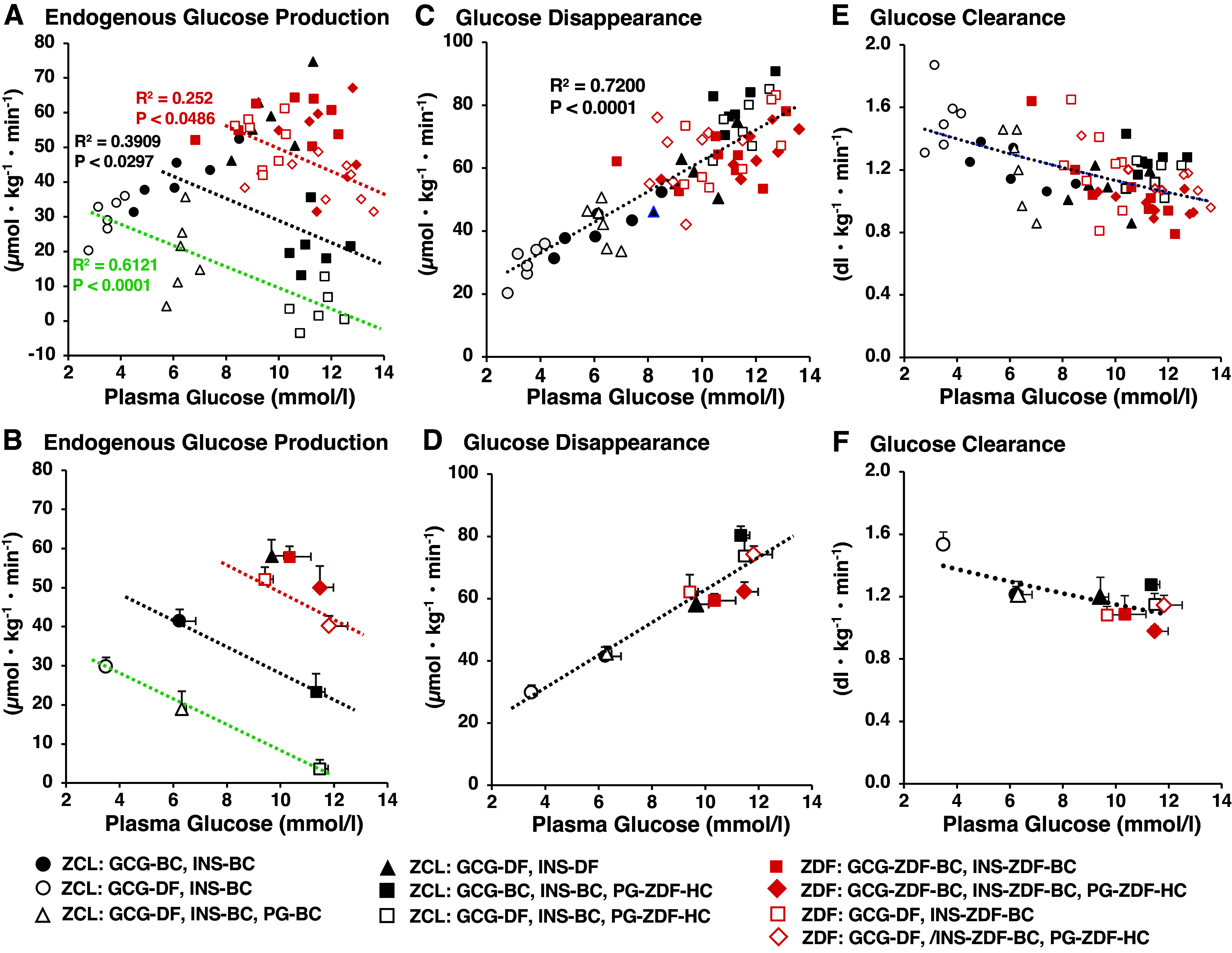
Effect of plasma glucose levels on endogenous glucose production, glucose disappearance, and glucose clearance rate under presence of basal insulin and glucagon, acute setting of glucagon deficiency, and combined glucagon and insulin deficiency in Zucker control lean rats (ZCL) and Zucker diabetic fatty (ZDF) rats. The symbols are defined as black solid circle, ZCL rats in the presence of basal insulin and glucagon (at 240 min in *protocol 1* in Fig. 1); black open circle, ZCL rats under glucagon deficiency (at 240 min in *protocol 2* in Fig. 1); black open triangle, ZCL rats under glucagon deficiency with euglycemic clamp in *phase II* (at 240 min in *protocol 3*, Fig. 1); black solid triangle, ZCL rats under insulin and glucagon deficiency (at 240 min in *protocol 4* in Fig. 1), black solid square, ZCL rats in basal glucagon and with basal ZDF glucose (hyperglycemic relative to basal ZCL) clamp (at 240 min in *protocol 5* in Fig. 1); black open square, ZCL rats in glucagon deficiency and with basal ZDF glucose (hyperglycemic relative to basal ZCL) clamp (at 240 min in *protocol 6* in Fig. 1); red solid square, ZDF rats in basal glucagon clamp (at 120 min in *protocol 7* in Fig. 1); red open square, ZDF rats in glucagon deficiency (at 120 min in *protocol 8* in Fig. 1); red solid diamond, ZDF rats in basal glucagon clamp and with basal ZDF glucose (hyperglycemic relative to basal ZCL) clamp (at 240 min in *protocol 7* in Fig. 1); and red open diamond, ZDF rats in glucagon deficiency and with basal ZDF glucose (hyperglycemic relative to basal ZCL) clamp (at 240 min in *protocol 8* in Fig. 1). Panels depict: correlation between endogenous glucose production and plasma glucose levels, and its statistical significance (*A*). Lines are defined as black line, under GGN-BC and insulin clamp at the basal plasma insulin of ZCL rats (INS-BC in ZCL rats) (black solid circle and black solid square); green line, under GGN-DF and INS-BC in ZCL rats (black open circle, black open triangle and black open square); red line, under GGN-DF in ZDF rats (red open square and red open diamond). *B*: means ± SE of plasma glucose concentration and endogenous glucose production of six animals in each group of ZCL rats and eight animals in each group of ZDF rats. *C*: correlation between glucose disappearance and plasma glucose levels, and its statistical significance. *D*: means ± SE of plasma glucose concentration and glucose disappearance of six animals in each group and eight animals in each group of ZDF rats. *E*: correlation between glucose clearance and plasma glucose levels. *F*: means ± SE of plasma glucose concentration and glucose clearance of six animals in each group and eight animals in each group of ZDF rats. GCG-BC, glucagon clamp at the basal plasma glucagon of ZCL rats; GCG-DF, glucagon deficiency; GCG-ZDF-BC, glucagon clamp at the basal plasma glucagon of ZDF rats; INS-BC, insulin clamp at the basal plasma insulin of ZCL rats; INS-ZDF-BC, insulin clamp at the basal plasma insulin of ZDF rats; PG-ZDF-HC, hyperglycemic clamp at the basal plasma glucose level of ZDF rats.

An elevation of PG acts on HGP as a negative feedback regulator (36). The relation between PG concentrations and EGP is summarized in Fig. 6, *A* and *B*. In ZCL rats, although in the presence of INS-BC, EGP correlated negatively with PG concentrations within the physiological range of its basal level regardless of the presence or absence of GCG-DF. GCG-DF shifted down the correlation between EGP and PG levels, suggesting that GCG-DF reinforces the effect of PG concentration to reduce EGP. INS-DF shifted up the relationship between EGP and PG levels under GCG-DF, suggesting that basal INS support the effect of PG concentration to reduce EGP. In ZDF rats, the relationship between PG concentration and EGP under either GCG-BC or GGN-DF in the presence of INS-BC are mostly above those of ZCL rats and close to that found under the combination of GCG-DF and INS-DF in ZCL rats, thus suggesting both basal glucagon and insulin having a limited impact on the relationship between EGP and PG concentration in ZDF rats.

PG concentration per se has potent effects on disposal of PG in periphery as well as the liver (36). The relation between PG concentrations and Rd is summarized in Fig. 6. Rd was tightly correlated positively with PG concentration (Fig. 6, *C* and *D*) with a similar glucose clearance (Fig. 6, *E* and *F*) regardless of the status of basal P-GCG and P-INS in ZCL rats, which was as observed in ZDF rats. This indicates PG concentration plays a dominant role for glucose disposal in periphery in both ZCL and ZDF rats. Glucose disposal in the postabsorptive state is known to be less dependent on insulin (38) and therefore, excessive postabsorptive HGP, rather than peripheral insulin resistance, may account for the hyperglycemia in ZDF rats.

## DISCUSSION

The present study demonstrates, under the postabsorptive state in healthy rats, the acute action of basal P-GCG substantially supports EGP and euglycemia by counteracting the acute action of basal P-INS, at least in part, via the cAMP signaling pathway in liver. We also show that inappropriately elevated P-GCG has a limited contribution toward the increased EGP and hyperglycemia in ZDF rats, an animal model of human T2DM-OB.

### Basal P-GCG Supports Postabsorptive HGP and Euglycemia in Healthy Rats

The contribution of basal P-GCG to support postabsorptive or fasting euglycemia has been extensively investigated in mice and rats with and without diabetes using a chronic blockade of glucagon signaling by treating with a GCGR inhibitor (24, 39–41), GCG gene deletion (42–50), α cell ablation (44, 47, 50, 51), or an immune-neutralization of P-GCG (47, 52), as well as an acute blockade of glucagon signaling by an immune-neutralization of P-GCG (53–61) (Table 3). Yet, the glucagon contribution remains indefinite, because *1*) wide variation in the effects reported after a blockade of glucagon, such as with a reduction of PG levels in healthy rodents ranging from 0 (no reduction) to 40% (Table 3); *2*) the effect of either a GCGR blockade or immune-neutralization of P-GCG to reduce HGP would suffer from negative feedback regulation, that counteracts the reduction of HGP from a loss of glucagon signaling by a reduction of P-INS and PG, which leads to the underestimation of the contribution of glucagon signaling to support postabsorptive HGP and euglycemia (42). In GCGR blockade via the treatment with GCGR monoclonal antibodies, elevation of plasma GLP-1 resulted as a side effect from the use of GCGR monoclonal antibodies, which stimulates GLP-1 secretion by L cells and would play a role in the reduction of HGP and PG levels, leading to the overestimation of the lowering of PG due to solely to a loss of glucagon signaling; and *3*) these studies with small animals did not assess the glucose flux associated with a change in blood glucose levels. In the present study using a pancreatic clamp method, we showed that in healthy rats, an acute setting of specific GCG-DF in the presence of INS-BC reduced postabsorptive EGP by 32% and consequently PG by 50%, suggesting that the acute action of basal P-GCG supports EGP and euglycemia in the postabsorptive state. Even with GCG-DF, an acute setting of INS-DF increased EGP and PG by 36 and 55%, respectively, compared with a setting of GCG-BC and INS-BC, which suggests a dominant role of basal P-INS to reduce EGP and PG. It is therefore likely that an acute action of basal P-GCG is to support EGP by counteracting the acute action of basal P-INS to reduce EGP.

**Table 3.** Effects of acute or chronic glucagon deficiency on blood glucose levels in rats and mice with health, obesity, or diabetes

Treatment				Animals								References
GCG Antibody	α-Cell Ablation	GCGR-KO	GCGR Blockade	Rats			Mice					
				Normal	STZ-T1D	ZDF	Normal	STZ-T1D	ob/ob	db/db	HFD-Fed	
Acute				↓ by 40% (F)								(53)
Acute				± (F)								(54, 55)
Acute				±								(53, 56)
Acute				↓ by 30%								(54)
Acute					±							(57)
Acute					± (F)							(58)
Acute					↓ by 40% (F)							(59)
		Chronic				↓ by 60%						(42)
		Chronic					↓ by 20–27%					(43, 44)
		Chronic					↓ by 25–40% (F)					(43, 45, 46, 48)
	Chronic						±-					(44, 51, 50)
	Chronic						± (F)					(51)
			Chronic				↓ by 40%					(39)
			Chronic				↓ by 27% (F)					(24)
		Chronic						↓ by 20–66%				(44, 48, 49)
Acute								↓ by 65%				(60)
Chronic	Chronic	Chronic						± (F)				(47)
Chronic									↓ by 43% (F)			(52)
Acute									↓ by 43% (F)			(61)
		Chronic								↓ by 26–66%		(42, 50)
		Chronic								± (F)		(50)
			Chronic								↓ by 25–40%	(39, 40)
			Chronic								± (F)	(41)
		Chronic									↓ by 25%	(44)

The symbols are defined as GCG antibody, neutralization of plasma glucagon by the treatment of glucagon antibody; ±, not changed significantly; ↓, reduction; (F), under fasting; Acute, outcome within 12 h after the treatment; Chronic, outcome after longer than a week with treatment; GCGR blockade, treatment with antibody against GCGR; GCGR-KO, reduced expression of glucagon receptor by deleting GCGR gene or treatment with GCGR-siRNA; STZ-T1D, type 1 diabetic model created by treatment with streptozotocin; ZDF, Zucker diabetic fatty rats; HFD-Fed, mice fed with high fat diet.

**Figure 7.**
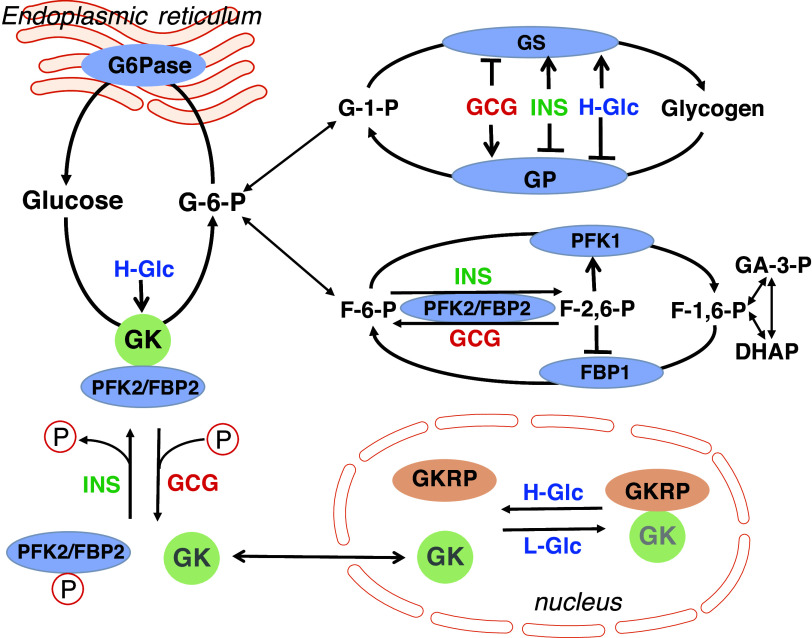
Involvement of the glucose&⇆glucose-6-phosphate (G-6-P), glucose-1-phosphate (G-1-P)&⇆glycogen cycle, and fructose-6-phosphate (F-6-P)&⇆fructose-1,6-bisphosphate (F-1,6-P)&⇆triose-phosphates cycles in the regulation of liver glucose flux by glucagon, insulin, and glucose. The direction and the rate of net hepatic glucose flux is determined by the relative activity of enzymes that catalyze nonequilibrium reaction in three substrate cycles; GS and GP in the G-1-P&⇆glycogen cycle, PFK-1 and FBP-1 in the F-6-P&⇆F-1,6-P cycle, and GK and G6Pase in the glucose/G6P cycle. In the glucose&⇆G-6-P cycle, GK catalyzes the phosphorylation of glucose to form G-6-P and G6Pase catalyzes the dephosphorylation of G-6-P to form glucose. During the past three decades, a mechanism, by which GK activity is acutely regulated, has been elucidated in studies using hepatocytes with enzymatic technology (62). Low glucose concentrations favor the binding of GK with GKRP, which promotes nuclear localization of GK and inhibits its catalytic activity. On the other hand, elevation of intracellular glucose concentrations promotes a dissociation of GK from GKRP which leads to a relocation of GK from the nucleus to the cytoplasm. A binding of GK with PFK-2/FBPase-2 supports a cytosolic localization and further increases the catalytic activity of GK. The binding is enhanced by dephosphorylation and reduced by phosphorylation of PFK-2/FBPase-2. In G-1-P&⇆glycogen cycling, GS attaches the glucose unit of UDP-glucose to a nonreducing end of glycogen and GP converts the glucose units into G-1-P by the breaking of α-4 bonds. In F-6-P&⇆F-1,6-P cycling, PFK-1 catalyzes the phosphorylation of F-6-P to F-1,6-BP and F1,6BPase catalyzes dephosphorylation of F-1,6-BP to F-6-P. F-2,6-P, a metabolite produced by the group of PFK2/FBP2, is an allosteric activator of PFK1 and inhibitor of FBP1. Activation of PKA by GCG via the cAMP signaling pathway promotes phosphorylation of GS, GP, and PFK2/FBP2; shifting net flux of G-1-P&⇆glycogen cycling, F-6-P&⇆F-1,6-P cycling and glucose&⇆G-6-P cycling toward glycogenolysis, gluconeogenesis and glucose production, respectively. Activation of PKB/Akt by INS via the PKB/Akt signaling pathway promotes the dephosphorylation of GS, GP, and PFK2/FBP2, shifting net flux of G-1-P&⇆glycogen cycling, F-6-P&⇆F-1,6-P cycling and glucose&⇆G-6-P cycle toward glycogenesis, glycolysis, and glucose utilization, respectively. DHAP dihydroxyacetone phosphate; FBP-1, fructose-1,6-bisphosphatase; GA-3-P, glyceraldehyde-3-phosphate; GCG, glucagon; GK, glucokinase; GKRP, glucokinase regulatory protein; GP, glycogen phosphorylase; GS, glycogen synthase; G6Pase, glucose-6-phosphatase; H-Glc, high concentration of glucose; INS, insulin; L-Glc, low concentration of glucose; PFK1, phosphofructokinase; PFK-2/FBPase-2, bifunctional enzyme.

The direction and the rate of net hepatic glucose flux are determined by the relative activity of enzymes that catalyze nonequilibrium reactions involving three substrate cycles: GS and GP in the glucose-1-phosphate/glycogen cycle, phosphofructokinase and fructose 1,6-bisphosphatase in the fructose 6-phosphate (F6P)&⇆ fructose-1,6-bisphosphate (F-1,6-P) cycle, and glucokinase (GK) and glucose 6-phosphatase (G6Pase) in the Glucose/G6P cycle (62). Recent studies at the cell or protein level propose a model that glucose, insulin, and glucagon regulate hepatic glucose flux by acting on these substrate cycles (Fig. 7). GK plays a critical role in the regulation of HGP by glucose and insulin. In the absence of glucose, GK in the superopened form has low intrinsic activity and binds the GK regulatory protein (GKRP) (63). The complex of GK/GKRP localizes to the nucleus in hepatocytes. An elevation of glucose concentration causes a concentration-dependent multiple step transition of GK to a closed conformation as it binds glucose to make GK active, not accessible for interaction with GKRP, and to translocate to the cytoplasm where it promotes glucose phosphorylation (64). In addition, insulin increases the Vmax value of GK by promoting the dephosphorylation of PFK-2/FBP-2 and the subsequent binding of GK with PFK-2/FBP-2 in the cytoplasm (65, 66). In the presence of INS-BC and GCG-BC, EGP correlated with PG concentration inversely (Fig. 6). The reduction of EGP with the elevation of PG was accompanied by a decrease in the EGP fraction contributing to TGO as well as an increase in GC and the GC fraction contributing to TGO without a change in TGO, suggesting that an elevation of PG decreased the provision of G6P from an endogenous source to the G6P pool. Therefore, basal P-INS is acting to reduce EGP by inhibiting glycogenolysis and/or gluconeogenesis, and simultaneously by increasing glucose phosphorylation (returning glucose to G6P). An acute setting of INS-DF increased EGP that was associated with an increase in TGO and the EGP fraction contributing to TGO, suggesting an increased provision of G6P from a endogenous source to the G6P pool. Despite elevated PG, GC and the GC fraction contributing to TGO were not increased, suggesting decreased glucose phosphorylation relative to the PG concentration. Thus, basal P-INS is acting to decrease endogenous G6P production and support glucose phosphorylation in response to PG. Glucagon is well known to stimulate glycogenolysis by activating GP and inactivating GS via the phosphorylation of these enzymes. In addition, glucagon promotes via cAMP signaling the phosphorylation of PFK-2/FBP-2, leading to the impairment of the interaction of this bifunctional enzyme with GK and to facilitate the GKRP-dependent nuclear sequestration of GK (66). In the presence of INS-BC, GCG-DF decreased EGP with a decrease in the EGP fraction contributing to TGO, while increasing GC and the GC fraction contributing to TGO. These results suggest basal P-GCG acts to maintain EGP by stimulating glycogenolysis and/or gluconeogenesis, not G6Pase activity, while simultaneously decreasing glucose phosphorylation (returning glucose back to G6P). Therefore, basal P-GCG acts to maintain basal EGP and PG levels by counteracting the acute action of insulin and glucose to reduce EGP and PG in healthy rats. The present study demonstrates that the resulting postabsorptive hepatic glucose flux, HGP, and GC are a consequence of the power balance among the acute actions of basal P-INS, PG concentration, and P-GCG on the substrate cycling pathways as shown in Fig. 7.

### ZDF Rats Fail to Respond to Changes in P-GCG to Regulate HGP, Demonstrating Severe Glucagon Resistance, in Addition to Insulin Resistance and Glucose Intolerance

Here, we provide the first evidence that acute selective GCG-DF exhibits a limited impact on postabsorptive EGP and hyperglycemia in ZDF rats. In ZCL rats, basal P-GCG contributes to the support of EGP and euglycemia via a cAMP signaling pathway in the liver. Compared with ZCL rats, hepatic cAMP levels in ZDF rats were not higher than those in ZCL rats, despite higher P-GCG levels in ZDF rats. However, unlike in ZCL rats, acute selective GCG-DF failed to reduce hepatic cAMP levels in ZDF rats. These results imply that the limited impact of P-GCG to regulate EGP and GC in ZDF rats results from the inability of basal glucagon to regulate intracellular cAMP levels.

Interestingly, despite the inability of P-GCG to regulate hepatic cAMP levels, basal levels of hepatic cAMP content in ZDF rats were maintained at the values seen in ZCL rats, raising the question of how basal cAMP levels and elevated EGP are sustained in rats displaying the characteristics of T2DM. It has been reported that the level of plasma catecholamines, which are able to activate adenylate cyclase via the β-adrenergic receptor in the liver, do not differ between that of ZDF and ZCL rats (67, 68). On the other hand, a known action of insulin is to activate cAMP phosphodiesterase activity (69). We reported previously that ZDF rats exhibit a compromised action of insulin and glucose effectiveness to reduce EGP by failing to inhibit glycogenolysis and activating GK (35, 70, 71). We demonstrate here that in ZCL rats, INS-DF elevates EGP and PG levels toward those seen in ZDF rats even with GCG-DF, suggesting a dominant role of insulin resistance to cause an elevation of EGP and hyperglycemia. Thus, hepatic resistance to insulin and glucose action may create a glucagon resistant state in ZDF rats. Since ZDF rats have severe insulin resistance (35, 71), the sustained levels of hepatic cAMP found in ZDF rats may result from resistance to this acute action of insulin.

### Increased GC in a Postabsorptive State in ZDF Rats May Not Be Associated with the Attenuated Glucagon Action on the Liver

Increased postabsorptive GC, a glucose flux alteration characteristically associated with humans having T2DM (72, 73), is also found in ZDF rats (28, 35, 74). The mechanism by which GC occurs at an increased rate in T2DM remains to be clarified. A possible mechanism might be a combination of the stimulation of glucose phosphorylation by hyperglycemia and a reduction in detritiation of [2-3H]-G6P as a result of a decrease in fructose-6-phosphate (F6P) &⇆ fructose-1,6-phosphate (F-1,6-P) &⇆ trios-P and pentose cycles in the liver. In the postabsorptive state, an increase in GC toward greater glucose phosphorylation in the liver would normally be associated with an increase in GK expression and/or sustained stimulation of the catalytic activity of GK by elevated PG levels. In the present study, we showed that the combination of GCG-DF and elevation of PG promoted a marked increase in GC in healthy rats. Therefore, one may doubt whether attenuated action of basal P-GCG on hepatic glucose flux and sustained hyperglycemia in ZDF rats are a possible factor for the increased GC in ZDF rats. However, this may not be the case. In the early and middle stages of diabetes in ZDF rats, 10 and 14 wk of age, respectively, GK expression is not higher when compared with their lean littermates, whereas the processes of GK activation in response to an elevated concentration of PG and P-INS, the dissociation of GK from the glucokinase regulatory protein, and the subsequent relocalization of GK from the nucleus to the cytoplasm, is impaired (28, 70, 71). In addition, normalization of hyperglycemia by treatment with a SGLT-2 inhibitor for 7 days before the clamp study normalized the increase in GC in response to hyperglycemia but did not alter the elevated basal GC (28, 35). Furthermore, at a late stage of diabetes in ZDF rats (∼20 wk of age), GC sustains at elevated level despite a marked reduction of GK expression (28, 74). It is therefore unlikely, that the elevated basal GC in ZDF rats at this late stage (∼20 wk of age) of diabetes is thereby associated with a function of GK in the liver. Alternatively, another possible explanation for the abnormal increase of GC might result from a decrease in fructose-6-phosphate (F6P) &⇆ fructose-1,6-phosphate (F-1,6-P) &⇆ trios-P cycle and pentose cycles that would decrease the detritiation of [3-^3^H]-G6P and consequently cause an elevation in [3-^3^H]-glucose specific activity in plasma, leading to a measurement that would suggest an increase in GC, since GC is calculated as the difference between [2-^3^H]-glucose Ra and [3-^3^H]-glucose Ra. However, it is not known whether the F6P &⇆ F-1,6-P &⇆ trios-P and pentose cycles in the liver are operating at three- to fourfold higher rates in ZDF rats compared with those in ZCL rats. Thus, we are not able to propose the true mechanism by which GC is increased in T2DM.

### A Limitation of Our Experimental Approach for Understanding Hepatic Glucose Fluxes

We adopted a one pool model for analyzing glucose flux in vivo with the tracer method. This model assumes one single pool of glucose metabolism in the body. Although there are three organs, liver, kidney, and small intestine, able to produce and utilize glucose in the body, the tracer method does not allow identification of the organ(s) where EGP, GC, and the actions of glucagon, insulin, and glucose occur. In healthy rats and humans, many of the parenchymal cells in the liver express the enzymes for glycolysis, glycogenolysis, and gluconeogenesis in every cell, while the relative activities of these enzymes differ in cells between the periportal to perivenous area in the liver lobules. GCGR is uniformly distributed in all parenchymal cells through the sinusoid of liver lobules (75, 76). Proximal tubular cells of the cortex in the kidney express all the enzymes for gluconeogenesis and can contribute toward ∼20–30% of EGP in postabsorptive rats (77) and 20% of EGP in overnight fasted humans (78). Since these cells do not express the appropriate hexokinase (HK) (79), GC may not take place in the kidney. In addition, gluconeogenesis (80) and cAMP signaling (81) in proximal tubules are insensitive to glucagon. The enterocytes in small intestine have a very limited level of G6Pase activity (82, 83) and release no glucose (82, 84) in rats fasted for shorter than 24 h. Thus, in healthy rats used in the present study, 70–80% of EGP and the alteration in EGP and GC resulting from GCG-DF may solely take place in the liver. In diabetic animal models and patients with T2DM, on the other hand, renal glucose production and its contribution to whole EGP have been reported to increase in the postabsorptive state (85). A marked increase in G6Pase activity in the small intestine has been reported in diabetic rats (82). The coexpression of G6Pase with HK in the enterocytes in the small intestine may allow GC to occur even under a lower blood glucose concentration due to the high glucose affinity of the HK. Although, the presence of glucose release from the small intestine in diabetic rats is still under debate (82, 84). In the present study, therefore, we could not conclude that the liver in ZDF rats is solely responsible for the elevated postabsorptive EGP and GC.

### Conclusions

The impact of basal plasma glucagon in the regulation of postabsorptive EGP and glycemia demonstrated in healthy rats using glucagon-insulin-glucose clamp techniques in the present study bears a striking resemblance to those reported in healthy humans using similar glucagon-insulin-glucose clamp techniques. Selective GCG-DF in the presence of INS-BC decreases EGP and blood glucose levels by 32 and 50%, respectively, in the present study versus 15–40% and 32–53%, respectively, in healthy humans (1–3). When levels of both PG and P-INS were kept at basal, selective GCG-DF reduces EGP by 60 and 75% in healthy rats and humans (4), respectively. When INS-DF is combined with GCG-DF, blood glucose and EGP increased by 36 and 55%, respectively, in healthy rats and 0–20% and 5–45%, respectively, in healthy overnight fasted humans (1, 4, 86, 87). Since basal plasma glucagon and insulin have a similar impact in the regulation of postabsorptive glucose metabolism in healthy humans and rats, rats may make a good model of humans for investigating the role of glucagon in metabolic regulation. This might be especially true for male ZDF rats, since they share many metabolic characteristics with humans with T2DM associated with obesity, such as hyperlipidemia, hyperglucagonemia, hyperinsulinemia, and hyperglycemia (25–28). ZDF rats develop nonalcoholic fatty liver disease (NAFLD) at a young age before the development of hyperglycemia (26–28). Recently, it has been reported that hyperglucagonemia correlates with NAFLD, not with altered glycemia in humans (9) and that the liver with NAFLD has glucagon resistance in regulation of amino acid metabolism (88). It has also been reported that adenylate cyclase activity, as well as glucagon-induced activation of this enzyme, are lower by 35–50% in hepatic plasma membranes from steatotic livers of humans (89) and that the increased cellular content of cholesterol and diacylglycerol that occurs in steatotic liver interferes with glucagon binding/activation of the GCGR (90), resulting in the uncoupling between GCGR and the Gs protein that appears to be mediated via activation of protein kinase C (91). Therefore, it is likely that an attenuation of the impact of glucagon to support postabsorptive endogenous glucose production and glycemia occurs in individuals with T2DM associated with obesity. Our results in rats suggest that the adoption of the pancreatic clamp method for investigating the role of basal P-GCG in the increased postabsorptive EGP and hyperglycemia in individuals with T2DM will yield similarly interesting results.

## DATA AVAILABILITY

The datasets used and/or analyzed during the current study are available from the corresponding author on reasonable request.

## GRANTS

This study was supported by a Grant DK-60667 from the National Institute of Diabetes and Digestive and Kidney Disease (NIDDK).

## DISCLOSURES

No conflicts of interest, financial or otherwise, are declared by the authors.

## AUTHOR CONTRIBUTIONS

M.S. conceived and designed research; S.K.E., C.S., T.P.O., and M.S. performed experiments; M.S. analyzed data; M.S. interpreted results of experiments; M.S. prepared figures; M.S. drafted manuscript; R.L.P. and M.S. edited and revised manuscript; S.K.E., C.S., T.P.O., R.L.P., and M.S. approved final version of manuscript.
